# Membrane Interactions Accelerate the Self-Aggregation of Huntingtin Exon 1 Fragments in a Polyglutamine Length-Dependent Manner

**DOI:** 10.3390/ijms22136725

**Published:** 2021-06-23

**Authors:** Arnaud Marquette, Christopher Aisenbrey, Burkhard Bechinger

**Affiliations:** 1 Chemistry Institute UMR7177, University of Strasbourg/CNRS, 67000 Strasbourg, France; marquette@unistra.fr (A.M.); aisenbrey@unistra.fr (C.A.); 2 Insitut Universitaire de France, 75005 Paris, France

**Keywords:** circular dichroism, dynamic light scattering, thioflavin T fluorescence, peptide-lipid interactions, huntingtin, Huntington’s disease, amyloid, htt17, membrane-driven aggregation

## Abstract

The accumulation of aggregated protein is a typical hallmark of many human neurodegenerative disorders, including polyglutamine-related diseases such as chorea Huntington. Misfolding of the amyloidogenic proteins gives rise to self-assembled complexes and fibres. The huntingtin protein is characterised by a segment of consecutive glutamines which, when exceeding ~ 37 residues, results in the occurrence of the disease. Furthermore, it has also been demonstrated that the 17-residue amino-terminal domain of the protein (htt17), located upstream of this polyglutamine tract, strongly correlates with aggregate formation and pathology. Here, we demonstrate that membrane interactions strongly accelerate the oligomerisation and β-amyloid fibril formation of htt17-polyglutamine segments. By using a combination of biophysical approaches, the kinetics of fibre formation is investigated and found to be strongly dependent on the presence of lipids, the length of the polyQ expansion, and the polypeptide-to-lipid ratio. Finally, the implications for therapeutic approaches are discussed.

## 1. Introduction

At least nine different hereditary diseases that are related to the expansion of a polyglutamine (polyQ) domain are known to date [1]. These so-called CAG (cytosine-adenine-guanine) repeat pathologies are all related by the propensity of their associated polypeptide to form insoluble β-sheet rich amyloid fibrils. Polyglutamine expansion promotes the self-assembly of fibrils and other types of aggregates that accumulate in inclusions found, for example, in the brain tissues of patients. By using modern imaging techniques, these have been described as dynamic phase-separated gel-like structures or as coexisting liquid/solid condensates [2,3]. Chorea Huntington is one of the best-studied polyglutamine-related diseases. In this case, it has been shown that the age of development and the severity of the disease correlates with the length of the polyQ stretch which is located within the amino-terminal domain of huntingtin, a protein encompassing about 3500 amino acids [1,4]. Symptoms of the disease develop when the polyQ expansion exceeds a critical length of ~ 37 glutamines [5,6]. Although the genetics of Huntington’s disease (HD) is well studied, the exact biological functions of huntingtin remain speculative, and the exact mechanism of pathogenic peptide aggregation remains a controversial topic [7].

It has been suggested that polyglutamines perturb neuronal membranes and result in their disruption concomitant with calcium dysregulation [8,9], thereby causing Huntington’s or other amyloidogenic diseases [10,11,12]. Huntingtin or fragments of the protein have been shown to be involved in intracellular vesicle trafficking [12,13,14]. They associate with the endoplasmatic reticulum, the Golgi-apparatus, and endosomal vesicles [8,13,15,16,17]. Notably, endosomal vesicle rupture has been shown to be a common mechanism in the redistribution between cells of large assemblies of α-synuclein, tau protein, and huntingtin [18]. Furthermore, the length of the polyglutamine tract of huntingtin affects the redistribution of these polypeptides between the cytoplasm and the nucleus [19,20,21]. Finally, a mitochondrial malfunction has also been associated with the pathogenesis of Huntington’s disease [22,23]. Indeed, when fusion proteins of glutathione S-transferase with exon 1 of huntingtin encompassing either 20 or 51 glutamines have been studied in association with brain lipid membranes, protein oligomerisation in the presence of long polyglutamine tracts is observed [24].

Studies on the various factors influencing the rate of aggregation include polyQ length, flanking sequences, posttranslational modification, protease activities on huntingtin, and the presence of chaperones [1]. On the one hand, the macromolecular assembly of huntingtin or its fragments through polyQ interactions is modulated by the association of the protein with membranes [12,25,26]. On the other hand, the length of the polyQ has an influence on these lipid interactions and the resulting membrane disruption [12,25]. Furthermore, the polyproline segment downstream of the polyQ domain has the opposite effect by reducing both the kinetics of aggregation and the formation of β-sheets by the polyQ region [25,27]. 

Importantly, the first 17 amino acids of huntingtin exon 1 (htt17, also abbreviated N17 or htt^N17^ by other authors), directly preceding the polyQ tract, and posttranslational modifications within this region have been shown to have a strong effect on the cellular localisation of huntingtin and the propensity of the protein to aggregate [8,27,28,29,30,31,32]. Thus, disease pathogenesis in transgenic mice can be inhibited by mutations of serine 13 and 16 within this htt17 domain [33]. Furthermore, the htt17 sequence carries phosphorylation, SUMOlation and nuclear export sequences [1,30,34,35,36,37,38]. Membrane interactions of huntingtin in vivo require this htt17 domain [8,15]. Furthermore, it has been shown that htt17 enhances polyglutamine oligomerisation [8,15,26,39,40,41] and is involved in the seeding and fibre maturation processes [26,42].

Recent structural investigations reveal high conformational plasticity of htt17 and the subsequent polyQ domain where htt17 association [26], its interactions with the membrane [43,44,45,46,47] or with other polypeptide domains are associated with random coil–helix structural transitions [48,49]. A recent NMR study has shown that in the solution, the htt17 sequence associates in a dimer of dimers preaggregation state [50,51]. By using electron paramagnetic resonance (EPR) labels positioned at different locations along the exon 1 sequence made up with 46 Qs, it was shown that the aggregation process is initiated by the *N*-terminal domain forming helical structures, followed by the polyQ adopting β-sheet conformations [26]. Membranes or seeds accelerate the aggregation process of this exon 1-Q46 construct. Notably in the solution, oligomers of 7–11 subunits have been observed [26], while it has been shown that a transition from coiled-coil α-helical super-secondary structures to β-sheet can be part of the aggregation process of polyQ proteins in general [52]. Molecular dynamics calculations indicate that β-hairpin containing conformers of polyQ probably act as templates for subsequent fibril formation [53,54]. The membrane-associated structure of the non-aggregating exon 1-Q25 variant has been investigated using EPR and Overhauser dynamic nuclear polarization approaches [44]. In the presence of membranes, the structuration reaches till residue 22, in agreement with a helical conformation covering much of the htt17 domain [55], while from residue 30 onward, the protein is water exposed and dynamic [44]. The htt17 domain insertion is shallow and reversible, with the helix axis parallel to the membrane surface [43,44,55,56]. 

Interestingly, the htt17 and the polyQ domains mutually influence each other and their conformational properties are coupled [57,58]. More recent investigations using electron microscopy and molecular dynamics calculations reveal tadpole-like structures of exon 1 where htt17, together with polyQ, forms a globular head domain increasing in size as the number of glutamines increases and where the polyproline extends as the tail [42,59,60]. The htt17 domain plays an important role in the polyQ aggregation, and *de novo*, seeded, or membrane-driven mechanisms have been distinguished [26]. Depending on the conditions, different aggregate sizes of globular or fibrous morphologies have been described and correlated to toxicity [17,61,62]. 

Aggregation, oligomer, and/or fibril formation which are the causative events for the development and progression of Huntington’s disease [17,61,62] require that polyglutamines are brought in contact with each other. This can occur by protein-mediated interactions of polyglutamines [1,63,64], by seeding, or by local accumulation of polyglutamines at bilayer surfaces [26,43,65]. Notably, the reversible membrane interactions of the amphipathic helical htt17 domain have been characterised in a quantitative and lipid-dependent manner [43] and the importance of this domain to enhance polyglutamine aggregation has been shown in vitro and in vivo [8,15,39,40,41]. Another huntingtin domain mediating membrane interactions has been identified within residues 171–371, a region bearing an overall high cationic character [12]. Furthermore, biochemical and cell biological assays demonstrate the potential role of these anchoring domains in disease development [12,24,45,46,56,66]. 

Membrane-surface-induced conformational changes in proteins play a critical role in the aggregation process, for example, by concentrating and aligning polyglutamines in such a manner to promote nucleation of amyloid formation [25,26,43,67,68]. Furthermore, membranes could alter aggregate morphology to specific toxic species or stabilise potentially toxic, transient aggregation intermediates [10,69,70,71]. Therefore, investigations of how amyloid fibrils as well as their intermediate and protofibrillar states interact with membranes is of considerable importance [25,72]. In the case of huntingtin and synuclein, it has been shown that fibrils are toxic to the cells [73] and that the docking of extracellular aggregates to the cell membranes is a key step of the vicious propagation–amplification cycle [74]. However, hardly any of these publications provide quantitative structural and biophysical data about interactions of huntingtin domains with membranes.

This prompted us to investigate in more detail the role of the membrane in the polyQ association kinetics. To this end, we prepared constructs involving the membrane-anchoring htt17 domain, followed by polyglutamines of different lengths. The structural changes were characterised in a time-dependent manner using circular dichroism (CD) spectroscopy and the supramolecular complexes formed by dynamic light scattering and ThT fluorescence. By investigating htt17 in the presence of polyQ domains as short as nine glutamines, the slower aggregation kinetics allowed for a more controlled evaluation of the processes involved in aggregation and fibril formation similar to the use of htt17-polyQ constructs used in previous biophysical studies (e.g., [45,46,51,59,66,75]). The polyglutamine was then successively extended in small steps to measure the effect of polyQs on the aggregation dynamics. The results reveal a pronounced dependence of the htt17-driven aggregation rates on the presence of lipid bilayers, the length of the polyglutamine tract, and polypeptide concentration. 

## 2. Results

The aggregation of huntingtin exon 1-derived peptides was studied by a combination of biophysical assays. The peptides encompass the first 17-residue amphipathic sequence known to reversibly interact with membranes [43] and polyglutamine stretches of variable length (Table 1).

### 2.1. Circular Dichroism (CD) Spectroscopy

**Figure 1:**
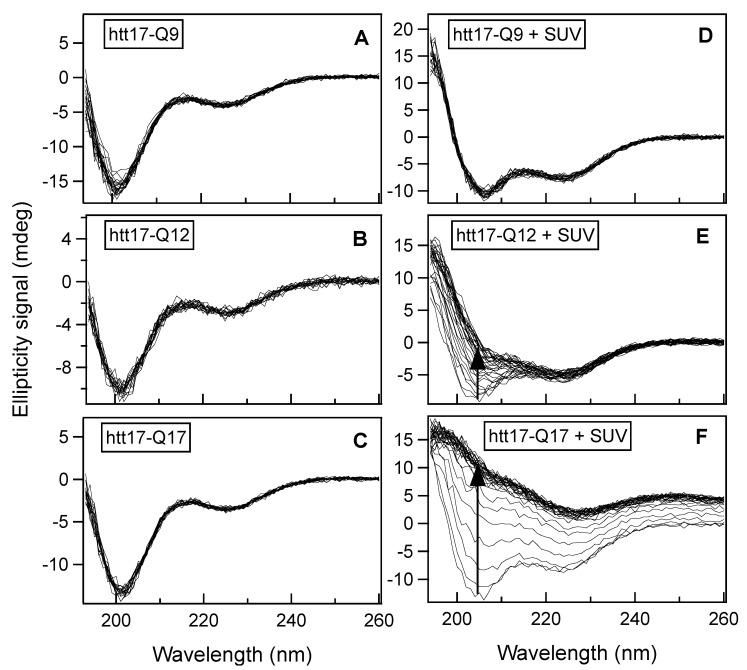
Time-dependent structural changes measured by circular dichroism: CD spectra of htt17-Q9, htt17-Q12 and htt17-Q17 (C = 9.1 × 10^−2^ mg/mL) in 10 mM Tris-HCl, pH 7 (**A**–**C**), and in presence of SUVs made of POPC/POPS 3/1 mole/mole (C = 0.45 mg/mL) (**D**–**F**) were recorded every 24.5 min. The progress of the spectral changes with time is depicted by arrows in panels **E** and **F**. Thereby the peptide-to-lipid ratios are 1/19.6, 1/22, and 1/26 for htt17-Q9, htt17-Q12, and htt17-Q17, respectively.

To test if membranes can increase the speed of polyglutamine aggregation and, at the same time, to gain insight into their secondary structure, we recorded CD spectra of the polyQ peptides htt17-Q9, htt17-Q12, and htt17-Q17 as a function of time. Their structural changes were monitored in the solution, in the absence (Figure 1A–C) and presence of phospholipid vesicles (Figure 1D–F) in 10 mM Tris-HCl, pH 7 at a concentration of 0.1 mg/mL (about 30 μM). The spectra were recorded between 260 and 194 nm in which the spectral line shape correlates with the secondary structure composition of the peptides. Measurements were performed every 24.5 min, and more than 18 spectra were recorded for each polypeptide sequence. 

In an aqueous solution, the three peptides all adopt predominantly random coil conformations, without any significant spectral changes over the time period of the experiment (Figure 1A–C). When fitting the data using the DicroProt analysis software [76], 64% of the signal are associated with random coil conformations. 

Figure 1D–F exhibits the time evolution of the peptide spectra in the presence of SUVs made of 1-palmitoyl-2-oleoyl-*sn*-glycero-3-phosphocholine (POPC)/ 1-palmitoyl-2-oleoyl-*sn*-glycero-3-phosphoserine (POPS) at a 3/1 molar ratio. The same mass of peptides (0.1 mg/mL) was mixed with a suspension of vesicles at a lipid concentration of 0.5 mg/mL in 10 mM Tris-HCl, pH 7, giving final peptide-to-lipid (P/L) molar ratios of 1/19.6, 1/22, and 1/26 for htt17-Q9, htt17-Q12, and htt17-Q17, respectively. Interestingly, the structure of htt17-Q9 remains unchanged over time since all the spectra overlap almost perfectly (Figure 1D). An estimate of the secondary structure of the peptide at the beginning of the experiment gives 33 % α-helix, 22% β-sheet, and 45% random-coil structures [76]. In contrast, the CD spectra, shown in Figure 1E, exhibit a significant increase in ellipticity over time, particularly between 194 and 220 nm, whereas the initial structure of the htt17-Q12 peptide resembles that of htt17-Q9 the β-sheet content of the Q12 sequence increases gradually, at the expense of α-helix and random-coil contributions. This effect is even more pronounced for htt17-Q17 in which the whole spectrum changes over time to converge ultimately to positive ellipticity values over the whole spectral range (Figure 1F). 

**Figure 2:**
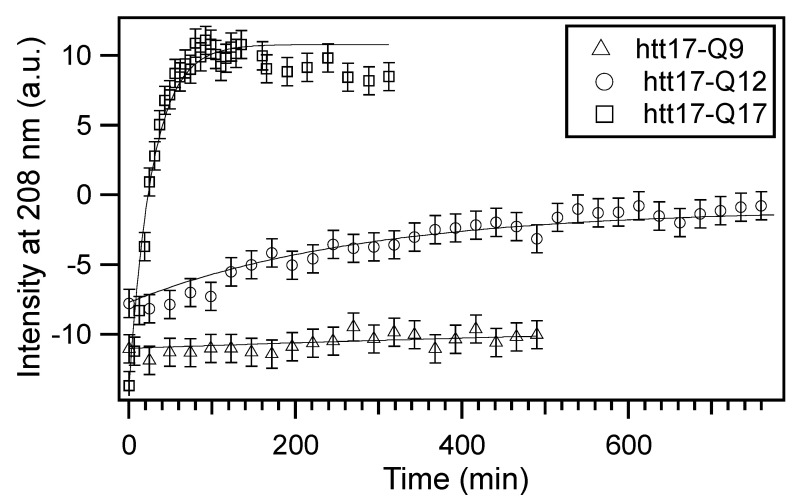
Aggregation kinetics by circular dichroism: the time-dependent intensity of the CD signal measured at 208 nm is shown for htt17-Q9, htt17-Q12, and htt17-Q17 in the presence of 0.45 mg/mL SUVs made of POPC/POPS 3/1 mole/mole in 10 mM Tris-HCl, pH 7. The results of least square fits with a mono-exponential function are displayed as solid lines. An error of ±1 was estimated from the signal-to-noise ratio of the spectra.

In order to characterise the kinetics of the peptide structural changes, we quantitatively analysed the time-dependent changes of ellipticity at 208 nm (Figure 2). The intensity at this wavelength shows the largest changes. Although it is often related to the helix secondary structures [77], it is also affected by vesicle aggregation processes [78]. A mono-exponential function of the form *A + B × (1–exp(-t/τ))*, where A and B are amplitude parameters and τ is the exponential time constant, describes well the intensity increase over time. The resulting fits are displayed in Figure 2. Although it should be noted that the absolute values are influenced by a number of environmental effects such as the stirring efficiency and the rate of dichroism increase, the aggregation rate 1/*τ* follows htt17-Q17 > htt17-Q12 > htt17-Q9 (Figure 2). This is confirmed by observations from longer polyglutamine peptides which aggregate too fast to be investigated by the techniques employed in this paper [78]. Furthermore, the peptides investigated here only aggregate in the presence of lipids within the time frame of the experiments.

### 2.2. Thioflavin T Fluorescence

To follow the kinetics of htt17-polyQ β-sheet formation, changes in the Thioflavin T (ThT) fluorescence were monitored in the presence of phospholipid bilayers. ThT is a popular reporter of amyloid aggregation because it demonstrates a strong shift and enhanced intensity of fluorescence emission upon binding to β-sheet rich fibrils [79,80]. Dye has been used to visualise and quantify the presence of misfolded protein or peptide aggregates in vitro and in vivo [81]. 

**Figure 3:**
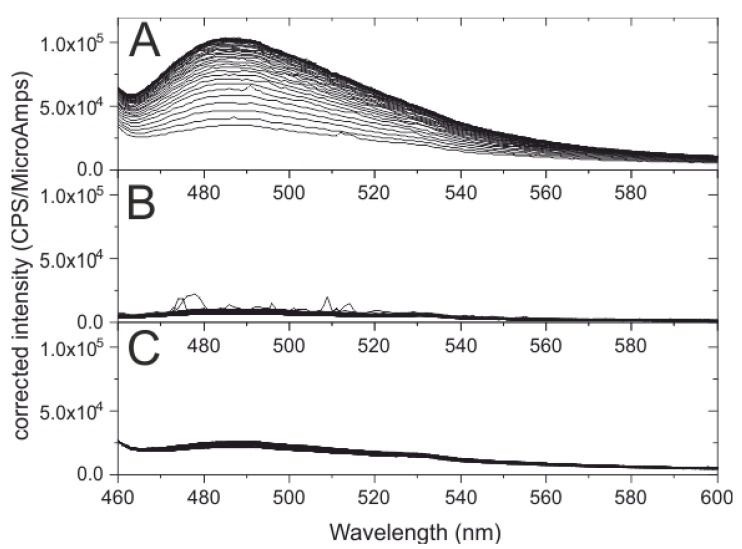
Time-dependent amyloid formation of htt17-Q17: the thioflavin T fluorescence is shown in the presence of 14.5 µM htt17-Q17 and SUVs made of 320 µM POPC/POPS 3/1 mole/mole in 10 mM Tris-HCl buffer, pH 7 (**A**). The peptide-to-lipid molar ratio was 1/22 and a spectrum was recorded every 2 min. The control experiments show almost no changes in thioflavin T fluorescence when exposed to the same amount of htt17-Q17 only (**B**) or SUVs only (**C**). The thioflavin T concentration was 5 µM in all recordings.

Figure 3 displays three sets of measurements performed at the same ThT concentration where a spectrum was recorded every 2 min over a time interval of 80 min. The intensity of the fluorescence emission spectra in 10 mM Tris-HCl buffer at pH 7 increases with time in the presence of both ≈14.5 µM htt17-Q17 and SUVs made of POPC/POPS 3/1 (Figure 3A). In contrast, the fluorescence measured in control experiments with peptides only (Figure 3B) or with SUVs only (Figure 3C) remains unchanged. In the presence of liposomes alone, an increase in the ThT fluorescence background suggests an interaction of the fluorophore with membranes (Figure 3C). 

In order to follow the β-sheet formation, we measured the time-dependent fluorescence intensity of ThT in the presence of the three peptides at the fixed wavelength of *λ_fluo_* = 485 nm, while the dye was continuously excited at *λ_exc._* = 440 nm. To quantify the effect of increasing peptide concentrations, for each htt17-polyQ sequence, three measurements were performed, namely, at P/L ratios of 1/88, 1/44, and 1/22. Under most experimental conditions, an increase of the signal was measured over time, indicating peptide aggregation. In some cases, the signal first increased and then decreased, which is due to sedimentation of the peptides, aggregated and/or associated with the vesicles [82]. Indeed, in these samples, precipitates were detected by visual inspection (Figure S1A) and fibrous structures made of peptide and associated lipids were observed by electron microscopy (EM) (Figure S1B–D). 

**Figure 4:**
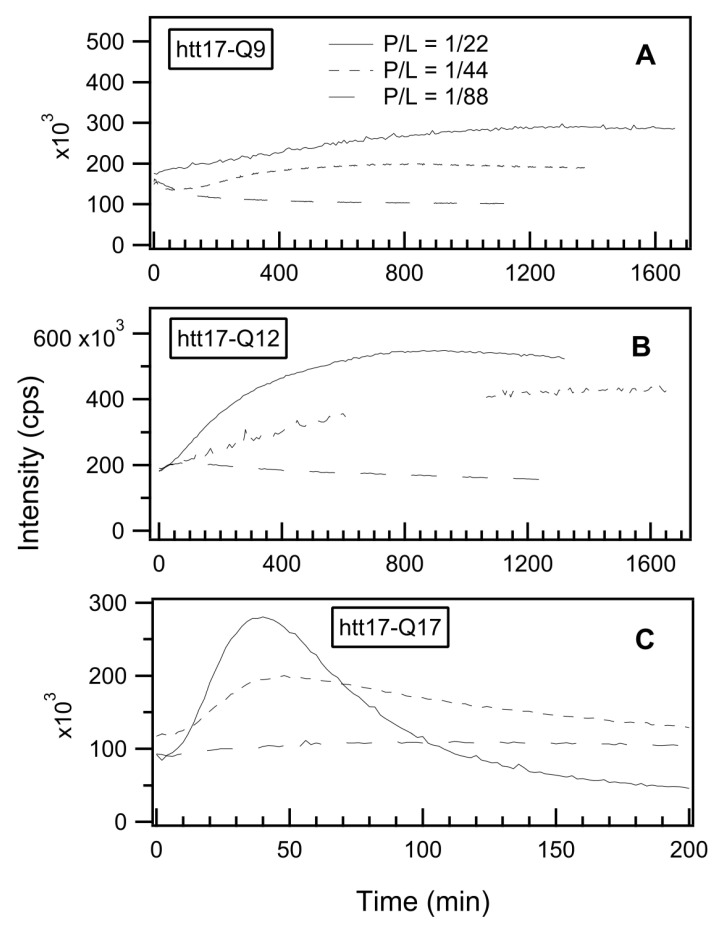
Time-dependent thioflavin T fluorescence as a function of polypeptide concentration: the thioflavin T fluorescence was continuously measured at 485 nm in the presence of SUVs and htt17-Q9 (**A**), htt17-Q12 (**B**), or htt17-Q17 (**C**), in 10 mM Tris-HCl, pH7. The peptide-to-lipid molar ratios are 1/22, 1/44, and 1/88, displayed as solid, short-dashed, and long-dashed lines, respectively. The lipid concentration was kept constant (C ≈ 320 µM) while the amount of peptide was adjusted to obtain the P/L ratios indicated. The ThT concentration was ≈ 5 µM in all the recordings.

As illustrated in Figure 4, the P/L ratio has a direct effect on the efficiency of the aggregation process as well as on its kinetics. For all three peptides, increasing the P/L ratio makes the aggregation process more efficient. Therefore, the fluorescence intensities measured at P/L = 1/22 are higher than for P/L = 1/44 or P/L = 1/88, while the kinetics (i.e., the τ values of the corresponding exponentials) are surprisingly similar when htt17-polyQ is investigated. In contrast, for P/L ratios 1/22, a mono-exponential signal increase describes the signal increase reasonably well, and additional processes initially cause negative slopes in the ThT fluorescence when htt17-Q9 is investigated at P/L ratios of 1/44 or 1/88, and for htt17-Q12 at P/L = 1/88. 

In order to test the reproducibility, the aggregation kinetics of htt17-Q17 in the presence of lipids (P/L 1/22) was tested six times by two different investigators and two different techniques. The τ values were 21 ± 3 min (*n* = 5) when determined by ThT fluorescence (Figure 3 and Figure 4C) and 29 min when the CD spectral changes were analysed (Figure 1F and Figure 2). For htt17-Q12, the time evolution was an order of magnitude slower (251 ± 83 min, *n* = 3; Figure 1E, Figure 2 and Figure 4B). 

### 2.3. Dynamic Light Scattering

**Figure 5:**
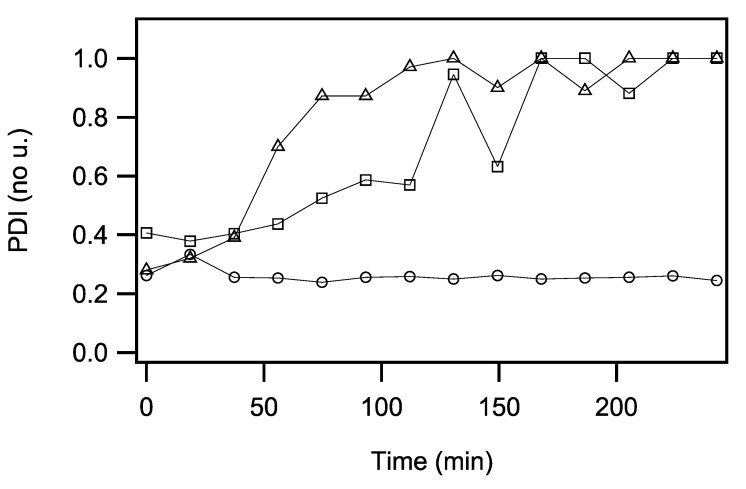
Time-dependent changes of polydispersity: the polydispersity index (PDI) is as a function of time for SUVs in the presence of htt17-Q9 (circles), htt17-Q12 (squares), or htt17-Q17 (triangles) in 10 mM Tris-HCl, pH7. Measurements were performed by dynamic light scattering in the absence of mechanical stirring. The same concentrations of lipids and peptides were used as in the CD experiments presented in Figure 1 and Figure 2. Data points were recorded every 19–20 min.

Dynamic light scattering measurements (DLS) were performed to obtain a more detailed view of the vesicle–vesicle interactions and the subsequent sedimentation processes that were suspected to occur in some of the fluorescence and CD spectroscopy experiments. In these experiments, the three htt17-polyQ peptides were exposed to suspensions of SUVs made of POPC/POPS 3/1 mole/mole (0.45 mg/mL, i.e., 580 μM) in 10 mM Tris-HCl, pH 7 buffer (Figure 5). To start with, the htt17-Q9 concentration was 29 μM and the hydrodynamic diameter of the molecular assemblies and their corresponding polydispersity indexes (PDI) were measured every 18.7 min for more than 7 h. Related experiments were performed in the presence of 25.9 μM htt17-Q12 and 22.9 μM htt17-Q17, respectively, i.e., the same concentrations by weight. This corresponds to the same experimental conditions also used for the CD measurements. When vesicles and htt17-Q17 are mixed, the DLS data indicate a rapid initial increase in polydispersity indicating fast aggregation and/or vesicle agglutination within 90 min. The changes of the PDI in the presence of htt17-Q12 occur at about half the pace. At the same time, the apparent hydrodynamic radius of the two systems also increases, but it should be kept in mind that the absolute value of this parameter is unreliable when the polydispersity approaches 1 (Figure 5). While the PDI and apparent hydrodynamic radii of htt17-Q12 and htt17-Q17 increase, the htt17-Q9 peptide leave the light scattering unaffected (Figure 5). Thereby, the apparent changes in ellipticity monitored in Figure 1 and Figure 2 were assigned to light scattering processes induced by supra-wavelength-sized systems such as vesicle and/or peptide clusters.

## 3. Discussion

Previously, it has been shown that huntingtin carries domains before and after the poly-Q tract that promote its reversible membrane association, and it has been suggested that this membrane association helps in polyQ aggregation [12,26,29,43]. In particular, the *N*-terminal 17 amino acids preceding the polyQ tract of huntingtin (usually abbreviated htt17 or N17 or htt^N17^) are involved in the regulation of the spatiotemporal distribution of huntingtin or fragments thereof. Their association with membrane components and with different cellular compartments has been shown to be important for the development of symptoms of Huntington’s disease [8,12,15,19,20,21,22,23,29,33,39,40,41]. Here, we show that htt17-polyQ membrane association indeed strongly accelerates polypeptide aggregation in a manner that is dependent on the number of glutamines. A series of sequences was investigated with htt17 as a membrane anchor and various polyQ extensions (Table 1).

In order to quantitatively measure the aggregation kinetics, biophysical experiments were performed at polypeptide concentrations of 3 to 30 μM. This relatively low concentration is still suitable for spectroscopic analysis, but longer polyQ constructs tend to aggregate at very short time scales [78]. Therefore, quantitative studies of the speed of aggregation were performed with constructs carrying a limiting number of glutamines (see also [83]). It should be noted that in their natural context the solubility of the full-length protein or its exon 1 domain is increased by the polyproline flanking sequence following the polyQ domain [31,32,40,48]. Therefore, as an alternative, exon 1 fragments were investigated where the carboxy-terminal proline-rich domain slows down the aggregation process, probably through interactions with polyQ and htt17 (e.g., [26,42,84]). As such additional interactions add an additional layer of complexity to the system, here we chose to present the role of htt17 membrane interactions in polyglutamine aggregation using shorter constructs that aggregate at a rate still accessible to the biophysical investigation. Furthermore, in the cellular environment, interactions with other proteins, chaperones, and proteases, as well as other domains of the full-length protein assure that healthy cells are protected from huntingtin aggregation [85,86]. Therefore, it takes many years to develop the disease even when much longer polyQ mutants are present [1].

When diluted in an aqueous buffer, CD spectra of the peptides with short polyQ additions are indicative of predominantly random coil structure and some features of α-helical/β-sheet conformations. Upon addition of POPC/POPS 3/1 membranes, the CD spectra of htt17-polyglutamines carrying 12 or more glutamines change in appearance over the next few hours (Figure 1). Notably, the CD spectra of htt17-Q17 exhibit similar features when compared to htt(1–40), a construct carrying htt17, 17 glutamines, and 6 prolines [52]. This observation suggests related structural changes in solution and in the presence of membranes. Electron microscopic images indicate that liposomes agglutinate with htt17-Q17 proteinaceous fibres (Figure S1B–D). The increase in PDI observed by DLS (Figure 5), the thioflavin fluorescence (Figure 3 and Figure 4), and the EM pictures (Figure S1) are indicative that the peptides aggregate in the presence of membranes in a manner that depends on the peptide-to-lipid ratio (Figure 4). In the case of htt17-Q17, the changes in the CD spectra obtained at concentrations of about 30 μM peptide and 650 μM lipid reach saturation after about 1.5 h (Figure 1 and Figure 2).

At longer incubation times, CD- and ThT fluorescence spectra decrease in intensity (Figure 2 and Figure 4C) because the larger peptide–lipid aggregates sink to the ground and/or stick to the surface of the glass tubes (Figure S1). The spectral changes are suggestive of an increase in β-sheet conformation (Figure 1), in agreement with other structural data [44,49,52,75] when, at the same time, a continuous increase in hydrodynamic radius is observed (Figure 5). Concomitant with this aggregation, a quantitative analysis of the resulting CD spectra is hampered by light diffraction artefacts (Figure 1F, Figure 2 and Figure 5). Peptides carrying a shorter polyQ segment aggregate more slowly or do not show spectral changes at all (Figure 1D,E). The degree of aggregation increases but also its kinetics accelerates with the number of glutamines.

In contrast to the peptides studied under the investigated conditions in this work, which remain in solution over many hours, even days in the absence of POPC/POPS vesicles, here, we show that the addition of membranes strongly catalyses the aggregation process. Indeed, whereas aggregate formation *de novo* or through seeding are well established pathways, membranes have been shown to provide a third aggregation mechanism [26,87]. The data presented agree with previous investigations where GST–exon1 constructs have been found to be associated with rat postsynaptic membranes and where brain lipid vesicles accelerate nucleation and thereby fibril formation upon trypsin cleavage of 3 μM GST–exon 1 encompassing 51 glutamines [24]. In the same study, the presence of zwitterionic DMPC or of DOPC/SM/cholesterol slowed down this effect, thereby being in line with studies where htt17 membrane interactions are weak or absent for zwitterionic and cholesterol-rich membranes [43,88]. In a related manner, a recent paper has revealed the importance of electrostatic contributions to the membrane association of htt17 within exon 1 and the possible interference of amino acid modifications introducing additional negative charges [44].

The *N*-terminal 17-residue sequence has been demonstrated to adopt a largely helical conformation when being membranes-associated [44,45,46,55,89], when part of a htt17-polyQ fibre [42,49,57,90] or in aggregation intermediates [62,83]. Here, we show that the membrane interactions of the htt17 flanking region of the polyQ domain promote the polyglutamine aggregation process. In a related manner, htt17 also plays a leading role in catalysing the nucleation process during polyQ aggregation in solution. The htt17 amphipathic helix has been shown to associate into small oligomeric structures which serve as nucleation sites [26,62,83] from which polyQ fibrils elongate [26,90,91]. Furthermore, the flanking sequences play important additional roles in regulating the proteolytic degradation of the protein and its aggregates where the toxicity depends in a complex manner on the conformational subpopulation rather than the polyQ aggregation propensity [30,32,62,92].

Flanking regions of other polyQ proteins seem equally important in the regulation of their aggregation and fibre formation [1]. In particular, previous studies showed that posttranslational modifications of flanking regions such as phosphorylation, SUMOylation, or ubiquitination including of htt17 have an effect on polyQ aggregation [1,30,34,35,36,37]. This has been attributed to the changes of htt17 oligomerisation being a consequence of such modifications, as well as modifications of its interaction surface with chaperones. The work presented here suggests that its interactions with membranes should also be strongly modified when, e.g., negative phosphates are attached to its serines [28], lysines are made unavailable for protein–protein and protein–lipid interactions [55,89,93], or the overall positive charge of htt17 is neutralised or inverted by posttranslational modifications, thus abolishing the electrostatic attraction of the flanking regions to anionic membranes [12,44,55]. The numerous possibilities to interfere with protein–protein and protein–lipid interactions and thereby fibre formation explain why not all polyQ-extension diseases follow the same pattern and why the age onset of Huntington disease shows significant variation even for the same number of glutamines [1].

We suggest that the amphipathic htt17 helix, which reversibly associates with the membrane interface [44,55,89], concentrates and aligns the polyQ chains in such a manner to facilitate intermolecular interactions and fibre association [12,43]. Once the interactions with the membrane are established, the aggregation process is enhanced by high peptide density (i.e., P/L ratio) and by extended polyQ chains but slows down by the presence of the polyproline stretch that follows the polyQ domain [27,48]. The polyQ length dependence may arise from the need to adopt an intermediate coiled-coil structure before transitioning into β-sheet amyloid [52] as well as from interactions with other proteins or polypeptide domains [1,25,27]. Therefore, these and other biophysical observations provide a rationale for a number of biochemical and cell biological experiments where huntingtin has been shown to be associated with membranes of intracellular organelles [8,12,15,19,20,21,22,23,28] or where the membrane anchoring domain htt17 has been demonstrated to promote the development of the disease [8,15,29,33,39,40,41].

Such lipid interactions have been shown to be modulators of aggregation and fibril formation also for α -synuclein [67,72,74], islet amyloid polypeptide [11], and β-amyloid [10,68]. Within these studies, the detailed membrane compositions including the resulting physicochemical properties such as negative charge density, fluidity, saturation, curvature, or interactions with specific lipids all play important roles in the aggregation process [11,43,44,66,88,94]. Therefore, the membrane interactions of polyQ flanking regions and their modulation by posttranslational modifications provide a possible therapeutic intervention site which has, to our knowledge, not been explored in greater detail.

## 4. Materials and Methods

### 4.1. Materials

All lipids were purchased from Avanti Polar Lipids (Alabaster, AL, USA). Water (HPLC grade), acetonitrile (99.8% HPLC grade), hexafluoro-2-propanol (99.5%), trifluoroacetic acid (TFA) (99.5%) and thioflavin (ThT) were from Sigma (St. Quentin Fallavier, France).

### 4.2. Peptide Synthesis and Purification

The peptides with the sequences shown in Table 1 were prepared by solid-phase synthesis using a Millipore 9050 automated peptide synthesiser and its standard Fmoc (9-fluorenylmethyloxycarbonyl) chemistry. They were purified by semi-preparative reverse-phase high-performance liquid chromatography (Gilson, Villiers-le-Bel, France) using a preparative C18 column (Luna, C18-100 Å-5µm, Phenomenex, Le Pecq, France) and an acetonitrile/water gradient. Their identity and purity (> 90%) were verified by analytical HPLC and MALDI mass spectrometry (MALDI-TOF Autoflex, Bruker Daltonics, Bremen, Germany). The fractions of interest were lyophilised for storage at −20°C. The peptide concentrations were determined by weighing several milligrams of powder at a high-precision laboratory scale (with 0.01 mg resolution). Although counterions were taken into consideration and care was taken to thoroughly dry the samples, systematic errors in the absolute concentrations of peptides may have arisen from residual salt and the hygroscopic nature of the sample.

### 4.3. Small Unilamellar Vesicles

The lipids were dissolved in chloroform/methanol (2/1 *v*/*v*). The solvent was evaporated under a flow of nitrogen gas in such a manner to form a homogeneous film on the walls of glass test tubes. The remaining solvent was removed by exposure for about 12 h to a high vacuum (*p* < 100 mPa). Subsequently, the lipids were hydrated in 10 mM Tris-HCl, pH 7 for more than an hour and subjected to several freeze–thaw cycles. Small unilamellar vesicles (SUV) were obtained after less than 1 min of tip sonication (Bandelin Sonopuls HD 200, Berlin, Germany). The lipid composition was chosen to represent the overall charge and main lipid components of the inner leaflet of the plasma membrane [95,96].

### 4.4. Circular Dichroism Spectroscopy

Circular dichroism (CD) spectra were recorded at 25 °C from 260 to 194 nm (spectral resolution: 1 nm, data pitch: 1 nm, scan speed: 100 nm/min) using a J-810 spectropolarimeter (Jasco, Tokyo, Japan). A total of 20 µL of peptide solution (at 1 mg/mL, in 10 mM Tris-HCl buffer pH 7) was transferred into a quartz cuvette of 1 mm path length and mixed with 200 µL of SUVs (C = 0.5 mg lipid/mL) to reach the final concentration of peptide ≈ 9.1 × 10^−2^ mg/mL (corresponding to 29, 26 and 22 μM for htt17-Q9, htt17-Q12 and htt17-Q17, respectively). The mixture was vortexed for ≈ 15 s prior to spectral acquisition. The secondary structure composition of the peptides was calculated from the spectra using a linear least-square method, implemented in the DicroProt analysis software [76].

### 4.5. Measurements of Thioflavin-T Fluorescence

A 100 mM stock solution of Thioflavin-T (ThT) was prepared in 10 mM Tris-HCl, pH 7, and stored in the dark at −20°C. The solution was thawed and diluted to 1 mM in the same buffer on the day of analysis. To prepare samples for fluorescence spectroscopy, peptide powders were suspended in some mL of mixed HFIP and TFA (1/1), dried, and dissolved in the same solvent at least three times in order to obtain a clear solution. Thereafter, for each sample preparation, an aliquot containing 0.3 mg of the peptide was taken and dried under a flow of N_2_ gas for more than 30 min. A few mL of SUV suspension (at 0.25 mg/mL) was added to the dried peptide to reach the appropriate peptide-to-lipid ratio, together with some µL of ThT solution, to reach a 5 µM final concentration. The solution was then vortexed for about one minute. Then, 1 mL of sample was rapidly transferred into a quartz cuvette and immediately placed in the sample holder of a Fluorolog 3–22 spectrometer (Horiba Jobin-Yvon, Longjumeau, France). The sample was excited at *λ_exc_* = 440 nm while the dispersed fluorescence intensity was either recorded from *λ_fluo_* = 460 to 600 nm or at the fixed wavelength of *λ_fluo_* = 485 nm at a constant temperature of 25 °C. A resolution of Δλ = 4 nm was chosen for excitation as well as for analysis in order to obtain a good signal-to-noise ratio. The sample was constantly stirred at low speed with a small size Teflon-coated magnetic bar.

### 4.6. Dynamic Light Scattering

Measurements were performed on a Zetasizer Nano-S system (Malvern Instruments, Worcestershire, UK) equipped with a 4 mW He-Ne laser. Samples containing the mixture of peptides and vesicles were placed in a low volume quartz cell equilibrated at 25 °C, and the light scattered backward was collected at an angle of *θ* = 173°. Data analysis was performed with the DTS Malvern software implemented on a personal computer.

### 4.7. Data Analysis

To analyse the kinetic data, i.e., the fluorescence intensity of ThT at 485 nm or circular dichroism at 208 nm as a function of time, a standard least-square fit analysis was implemented numerically. A three-parameter single exponential function *I*(*t*) *= A + B × (1–exp(-t/*τ*))* was found to describe well the intensity increase of the signals.

## Figures and Tables

**Table 1:** Amino acid sequences of huntingtin exon 1-related peptides (UniProt P42858).

htt17-Q17	MATLEKLMKAFESLKSF QQQ QQQ QQQ QQQ QQQ QQ
htt17-Q12	MATLEKLMKAFESLKSF QQQ QQQ QQQ QQQ
htt17-Q9	MATLEKLMKAFESLKSFQQQ QQQ QQQ

## Data Availability

Data are available on request.

## Supplementary Materials

The following are available online at https://www.mdpi.com/article/10.3390/ijms22136725/s1, Figure S1: Picture and TEM image of htt17-Q17 in the presence of 100 nm LUVs.
