# Supplementary material for: Membrane Interactions Accelerate the Self-Aggregation of Huntingtin Exon 1 Fragments in a Polyglutamine Length-Dependent Manner
Source: Int J Mol Sci. 2021 Jun 23;22(13):6725. doi: 10.3390/ijms22136725 (PMC8268948; doi:10.3390/ijms22136725)
Supplement: Supplementary file 1 [file ijms-22-06725-s001.zip › ijms-1241073-supplementary.pdf]

SUPPLEMENTARY INFORMATION

Membrane interactions accelerate the self-aggregation of huntingtin exon 1 fragments in a polyglutamine length-dependent manner

A. Marquette, C. Aisenbrey and B. Bechinger*

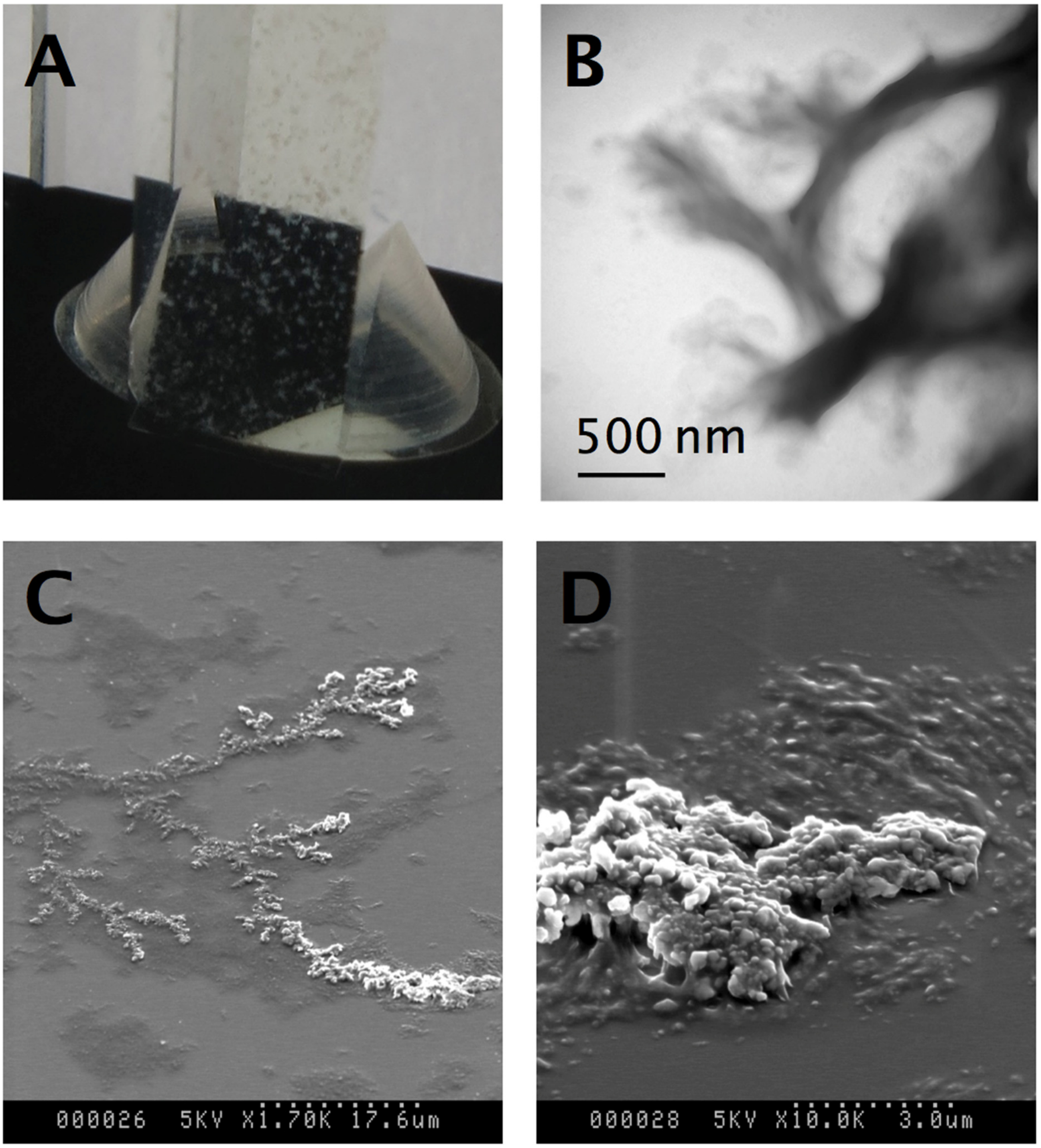

Figure S1 shows images of htt17-Q17 in the presence of 100 nm LUVs made of POPC/POPS 3/1 mole/mole (P/L = 1/26). **A.** Picture of a sample used for ThT fluorescence experiments (Figure 4) and taken after more than 1 hour of measurements. The flocculation of

lipid/peptide structures was visible in the cuvette under eye examination. **B**. The peptide-lipid mixture has been applied onto a copper grid and investigated by transmission electron microscopy. Bundles of fibers (dark) with liposomes attached (light grey) can be distinguished (enlargement 60000 x). **C** and **D**. Scanning electron microscopy images of the same preparation deposited onto a small size glass plate at 1700 x and 10000 x magnification, respectively. The images are suggestive of vesicles aligned along linear branched structures.
